# Individualized Monitoring of Muscle Recovery in Elite Badminton

**DOI:** 10.3389/fphys.2019.00778

**Published:** 2019-06-26

**Authors:** Vanessa Barth, Hannes Käsbauer, Alexander Ferrauti, Michael Kellmann, Mark Pfeiffer, Anne Hecksteden, Tim Meyer

**Affiliations:** ^1^Institute of Sports and Preventive Medicine, Saarland University, Saarbrücken, Germany; ^2^German Badminton Association, Olympic Training Center, Saarbrücken, Germany; ^3^Faculty of Sports Science, Ruhr University Bochum, Bochum, Germany; ^4^Institute of Sports Science, Johannes Gutenberg University Mainz, Mainz, Germany

**Keywords:** reference range, Bayesian, fatigue, sport, recovery

## Abstract

**Purpose:** Individualized reference ranges for serum creatine kinase (CK) and urea are a promising tool for the assessment of recovery status in high-level endurance athletes. In this study, we investigated the application of this approach in racket sports, specifically for the monitoring of elite badminton players during the preparation for their world championships.

**Methods:** Seventeen elite badminton players were enrolled of which 15 could be included in the final analysis. Repeated measurements of CK and urea at recovered (R) and non-recovered (NR) time points were used for the stepwise individualization of group-based, prior reference ranges as well as for the evaluation of classificatory performance. Specifically, blood samples were collected in the morning following a day off (R) or following four consecutive training days (NR), respectively. Group based reference ranges were derived from the same data. Error rates were compared between the group-based and individualized approaches using the Fisher exact test.

**Results:** Error rates were numerically lower for the individualized as compared to the group-based approach in all cases. Improvements reached statistical significance for urea (test-pass error rate: *p* = 0.007; test-fail error rate: *p* = 0.002) but not for CK (*p* vs. group-based: test-pass error rate: *p* = 0.275, test-fail error rate: *p* = 0.291). Regardless of the chosen approach, the use of CK was associated with lower error rates as compared to urea.

**Conclusion and Practical Applications:** Individualized reference ranges seem to offer diagnostic benefits in the monitoring of muscle recovery in elite badminton. The lack of significant improvements in error rates for CK is likely due to the large difference between R and NR for this parameter with error rates that are already low for the group-based approach.

## Introduction

In elite sport, the assessment of recovery status has become an important goal to prevent accumulating fatigue which may lead to maladaptive states such as non-functional overreaching or overtraining and increase the risk of injury. During the last decades numerous fatigue indicators have been described including (but not limited to) subjective ratings, heart rate measures as well as blood borne markers (Majumdar et al., 1997; Hecksteden et al., 2016, 2017). However, large interindividual variability impedes on the diagnostic accuracy of all fatigue indicators known to date and thereby limits their utility in the assessment of individual athletes (Majumdar et al., 1997; Hecksteden et al., 2016). Individualized reference ranges may offer a solution to this challenge, comparable to the principle of the Athlete Biological Passport (Sottas et al., 2007; Hecksteden et al., 2016). Following this rationale, our group has recently developed a method to gradually individualize group-based (prior) reference ranges of fatigue indicators (Kellmann et al., 2018) resulting in separate “corridors” for recovered (R) and non-recovered (NR) states. In this work we aim to scrutinize transferability of this method to elite badminton by monitoring the preparation phase of one part of the German national team for the world championships 2017.

Following this rationale, we opted to employ the same markers and very similar methodology which have been successfully used in the original publication of the method (Kellmann et al., 2018). Blood borne markers seem particularly promising due to their objectivity, minimal interference with the training process, low technical error of measurement and known physiology (Majumdar et al., 1997; Hecksteden et al., 2017). Among the multitude of blood borne fatigue indicators (Majumdar et al., 1997; Hecksteden et al., 2017), creatine kinase (CK) and urea have been selected for the development of the individualization procedure (Kellmann et al., 2018) as well as for this work for several reasons. From a practical perspective, CK and urea are inexpensive to measure and already known in sports practice. From the physiological perspective, urea as the end product of protein breakdown reflects metabolic strain and ultimately energy balance. It is therefore mainly elevated by high training volumes (Hecksteden et al., 2017). By contrast, serum CK levels increase especially after eccentric muscle contractions, therefore CK is widely used as a marker of training induced muscle strain and recovery. It may therefore be plausibly expected to play an important role in badminton. The appropriateness of the selected markers for badminton is underlined by the previously reported increase in serum CK and urea levels 12 h after badminton specific training (Sottas et al., 2011). However, it has to be kept in mind that the applicability of the individualization algorithm is in principle not limited to CK and urea or even to blood borne markers in general.

Taken together, we report the first application of a recently published method for the individualized monitoring of muscle recovery in elite sports practice, specifically the preparation of badminton players for world championships. We thereby scrutinize the method’s transferability from endurance to racket sports as well as into sports practice.

## Materials and Methods

### Experimental Approach to the Problem

**FIGURE 1:**
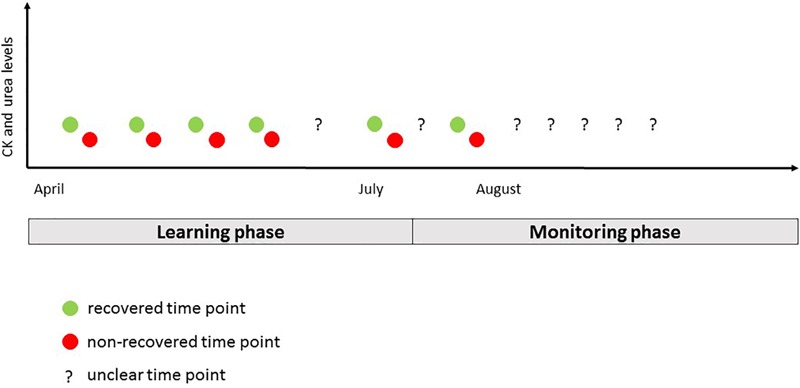
General design.

The present work employed an observational approach. Due to the importance of the competitive event, any interference with the training process would have been unacceptable for coaches and athletes. As illustrated in Figure 1, the study period from April to August 2017 included a learning phase for the derivation of individualized reference ranges (April–early July) and an individualized monitoring phase during the immediate preparation for the world championships (Mid-July–August). According to the published algorithm (Kellmann et al., 2018), individualized reference ranges were derived from values for CK and urea at time points with known recovery status (R or NR, respectively). Considering that a reference classification is needed for the assessment of error rates, time points from the learning phase were also used to assess the performance of the individualization procedure (Kellmann et al., 2018). Of course, this was conducted as a cross-validation, meaning that the data points to be classified were not included in the respective run of the individualization procedure. Therefore, the main results of this work are based on data from the learning phase. Results of the individual monitoring phase are presented graphically to illustrate the potential application in sports practice.

### Subjects

Seventeen elite male badminton players, all members of the German national squad and training at the National Training Center of the German Badminton Association and Olympic Training Center Rheinland-Pfalz/Saarland in Saarbrücken, volunteered to participate in this study. Females could not be included because they were training in another center. Each participant was informed about the experimental procedures of the study and provided written informed consent. The study was approved by the local Human Research Ethics Committee (Ärztekammer des Saarlandes, approval no. 228/13 and amendments). Two players had to be excluded due to an insufficient number of time points during the learning phase. Characteristics of the remaining 15 athletes are summarized in Table 1.

**Table 1:** Subject characteristics.

Age (years)	22 ± 3
Height (cm)	183 ± 6
Weight (kg)	78 ± 9
Years playing competitive badminton	10 ± 4
Training volume (h/week)	22 ± 2

*Means ± standard deviation.*

### Procedures

Venous blood samples were collected in the morning before the first training bout of the day. Standard methods were used for venous blood sampling and analysis as previously published (Hecksteden et al., 2017). In particular, CK and urea were analyzed within 60 min by automated routine techniques (UniCell DxC 600 Synchron; Beckman Coulter GmbH, Krefeld, Germany).

Procedures and statistical analyses were conducted in analogy to the initial publication of the method as described in short below.

#### Reference Classification of Time Points During the Learning Phase

**FIGURE 2:**
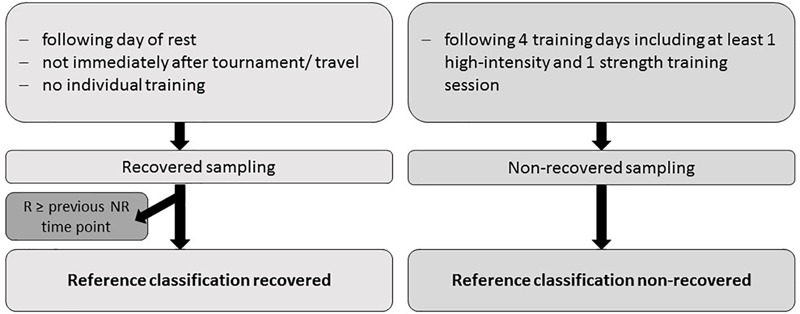
Reference classification of time points. R, recovered; NR, non-recovered.

**FIGURE 3:**
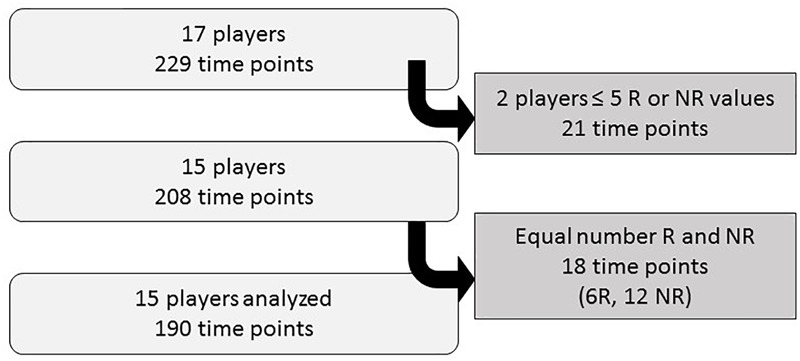
Criteria for data inclusion. R, recovered; NR, non-recovered.

In the learning phase, values for serum CK and urea were obtained during competition-free weeks and adapted to the individual trainings plans of the players. Criteria for the reference classification of R and NR time points are summarized in Figure 2. The resulting numbers of time points and players are illustrated in Figure 3. Training load and the possible presence of other physical loads was checked for every individual player by reviewing training logs and standardized questions. Personal communication was sought when needed. Additionally, a validated questionnaire (Acute Recovery and Stress Scale, ARSS; Sottas et al., 2007; Kellmann et al., 2016; Kellmann and Kölling, 2019) was completed by each participant before blood sampling to verify subjectively perceived changes in recovery status. The German version of the ARSS, which was used in this study, contains eight items describing physical, emotional, mental and overall aspects of recovery and fatigue using a seven-point scale.

During the individual monitoring phase blood sampling was conducted on request of the coaches at the same time of day as during the learning phase.

#### The Individualization Procedure

Details of the individualization procedure including equations, statistical code and sport specific prior distributions have been published previously (Kellmann et al., 2018). In short, for every parameter a group based (prior) distribution is used as starting point. Importantly, relevant differences in the distribution of CK and urea between different sports were not present (Kellmann et al., 2018). Subsequently, individualized (posterior) distributions for NR and R time points are generated by stepwise inclusion of measurements from the individual athlete in the respective state.

**FIGURE 4:**
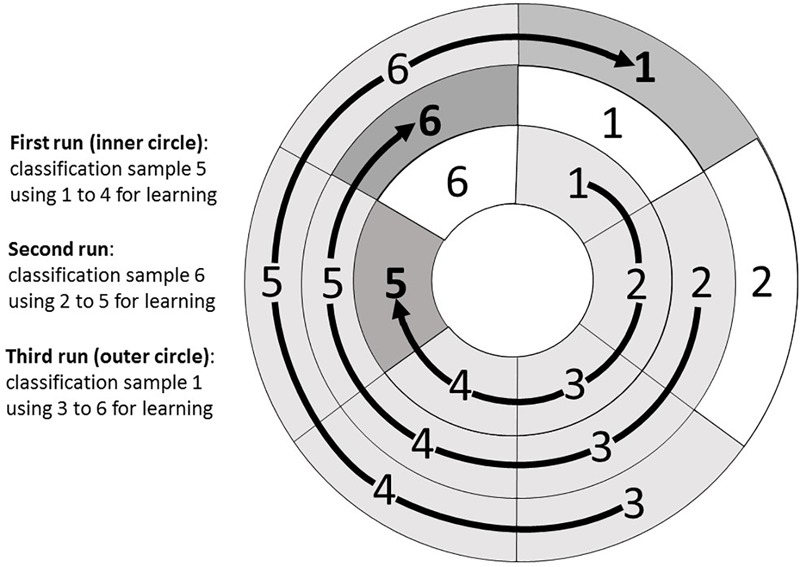
Runs of the individualization procedure (exemplarily for six time points). For every run 4 recovered (R) and 4 non-recovered (NR) values were used in chronological order for the deduction of the individualized cut-off value which was then used to classify the following R and NR values. Values to be classified are marked dark gray, samples for learning are marked light gray.

For every subject several runs of the individualization procedure (equal to the number time points per fatigue state) are conducted to allow for the classification of all time points. For every run 4 R and 4 NR values were used in chronological order for the deduction of the individualized cut-off value which was then used to classify the following R and NR values, respectively. The principle is illustrated in Figure 4.

**FIGURE 5:**
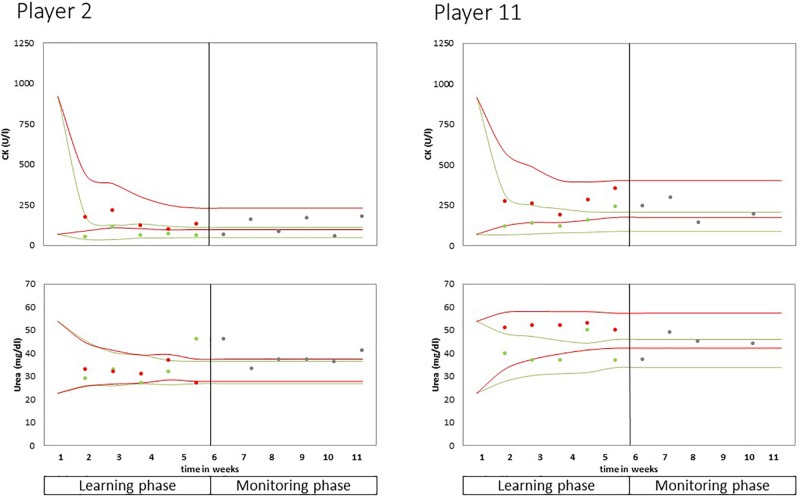
Individual distributions of CK and urea levels.

The principle of the stepwise individualization procedure is illustrated in Figure 5 which shows the development of the individual “corridors” for one of the athletes. Of note, Figure 5 also includes the values from the individual monitoring phase.

#### Classification of Time Points and Calculation of Error Rates

The cut off value for the individualized classification of time points was set at equal distance between the posterior means for R and NR time points as resulting from the forth individualization step. Importantly, no cut-off value was calculated when the posterior mean for R was higher than the posterior mean for NR after the forth individualization step. Rather, the respective run was excluded because the fifth value was considered non-classifiable (Kellmann et al., 2018).

The group-based comparator classification was based on the overall mean for the respective parameter (which, due to the equal number of time points between states is located at equal distance to the group means for R and NR time points). Thereby, the group-based cut-off value was based on the same data that were to be classified to avoid overestimating a potential benefit of the individualization procedure.

Error rates of the individualized and group-based approaches were determined by comparing classifications based on CK and urea with the reference classification. The proportion of data points that were falsely classified as R among all R classifications was defined as test-pass error rate. Test-fail error rate was defined as the proportion of data points falsely classified as NR among all NR classifications.

### Statistical Analyses

All statistical analyses were conducted using Statistica software version 13.3 (StatSoft Hamburg). Raw values for CK as well as urea were log-transformed before any further calculations. The log-transformed data were normally distributed for either parameter. Results were transformed back to the original scale. Descriptive statistics are reported as means and standard deviations (SD). Differences in CK and urea between R and NR time points were verified on the group level using a mixed linear model analysis. Recovery status was included as a fixed effect, the player’s identity and status-by-subject ID interaction were random effects. To analyze the differences in error rates between the individual and the group-based classifications a Fisher’s Exact test was conducted. The level of significance was set with an α-error of *p* < 0.05 for all tests.

## Results

**FIGURE 6:**
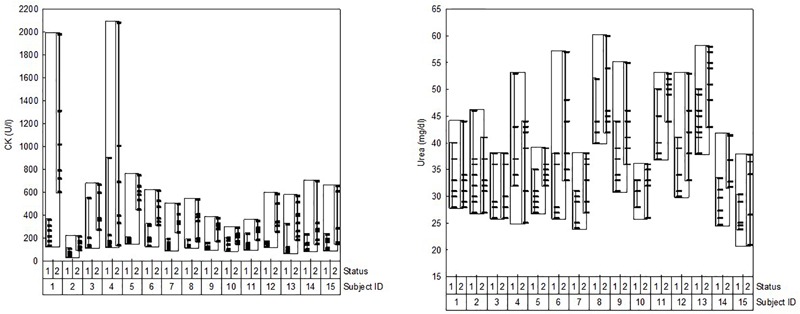
Intra- and individual variabilities of CK and urea. Status: 1 = recovered, 2 = non-recovered.

Mean values for CK and urea were significantly higher for the NR compared to the R time points (CK (U/l): R 164 ± 106, NR 425 ± 319, *p* < 0.001; urea (mg/dl): R 35 ± 7, NR 39 ± 9, *p* < 0.001). As illustrated in the respective variability plots (Figure 6), considerable interindividual variation could be observed for individual mean values as well as for the difference between R and NR states.

Error rates were numerically lower for the individualized as compared to the group-based classification for either parameter and recovery status. However, the difference reached statistical significance only for urea (Table 2). Importantly, absolute values for CK error rates were already low for the group-based approach due to the large effect size of the difference between R and NR time points.

**Table 2:** Test-pass and test-fail error rates for the group-based and the individualized classification.

	Group-based	Individualized	*p*
**CK (U/l)**			
Test-pass error rates	15 (16%)	9 (10%)	0.2751
Test-fail-error rates	24 (25%)	17 (18%)	0.2907
**Urea (mg/dl)**		
Test-pass error rates	41 (43%)	19 (23%)	<0.01
Test-fail-error rates	44 (46%)	19 (23%)	<0.01

*Test-pass error rate was defined as the proportion of data points that were falsely classified as recovered among all recovered classifications. Test-fail error rate was defined as the proportion of data points falsely classified as non-recovered among all non-recovered classifications.*

The rate of unclassifiable values was 15% (*n* = 28) for urea and 2% (*n* = 4) for CK.

The development of individual corridors over the learning phase as well as measured values from the individual monitoring phase are displayed in Figure 5. In this figure, player 2 and 11 are used as examples to illustrate the interindividual differences between reference ranges: CK values of 250 U/l are at the upper range of the NR corridor of player 2, whereas in player 11, this value can be found in the lower NR range. Urea levels do hardly differ between the R and NR states in player 2, but in player 11, urea levels are situated between 30 and 40 mg/dl when the player is recovered and about 50 mg/dl for the NR state. Of note, the urea corridors of player 11 show that values exceeding the clinical reference limit can be the physiological level for a specific, healthy individual. The corridors of each player are provided in Supplementary Figure S1. The individualized monitoring in this structured two-step procedure and particularly the visualization of reference ranges as individual corridors (Figure 5 and Supplementary Figure S1) were evaluated as very helpful by the coaches (oral communication).

## Discussion

Reduced error rates for the assessment of muscle recovery in elite badminton from the use of a novel procedure are the main result of this study. Individualized reference ranges being derived from time points with known recovery status during the learning phase can be used for the individualized monitoring of athletes during the decisive training phase preceding the event. Although “success” of the individual monitoring phase may not be formally quantified within the framework conditions of the present trial, the individualized monitoring was deemed very helpful by the coaches. Together our results point to the transferability of individualized reference ranges for CK and urea to the monitoring of muscle recovery in elite sports practice, specifically in racket sports.

Regarding the magnitude of improvement in diagnostic accuracy, considerable differences between parameters were observed. While for urea error rates could be almost reduced by 50%, for CK the reduction in error rates (although numerically present) was less pronounced and failed to reach statistical significance. When interpreting the differences in the effect of individualization it should be kept in mind that group-based error rates for CK were already much lower compared to urea, leaving little potential for improvement. The lower group-based error rates for CK are attributable to the large overall CK response in our data which is readily explained by the high proportion of muscular strain in badminton due to the frequent accelerations and decelerations. Obviously, a larger mean difference between states and lower within-state variation both improve diagnostic accuracy of the respective indicator. This consideration also pertains to the higher rate of unclassifiable values for urea as compared to CK.

Moreover, the principle that the usefulness of CK and urea, respectively, for monitoring muscle recovery depends on the relationship of between-state contrast and within-state variation (“individual effect size”) also applies on the individual level. Importantly, the variability plots displayed in Figure 6 illustrate that athletes greatly differ in this respect. The calculation of individualized normal ranges for R and NR time points accounts for the variable between-state contrast by providing an individualized two point calibration of the respective marker. In other words: Measured values are interpreted in relation to two individualized anchor points which represent the extremes of recovery status attained during habitual training cycles. As a result, inferences from a measured CK or urea value on the current recovery status of a particular athlete may be made more confidently as compared to approaches which rely on only one (even if individual) reference. The same applies for interpreting the magnitude of changes in those markers. Taken together, the method applied in this work accounts for two challenging characteristics of blood-borne fatigue markers which so far impede on the assessment of recovery status in athletes (Figure 6): (i) differences in habitual levels and (ii) differences in the magnitude of changes in fatigue markers between R and NR time points. While the method employed here is the first one to offer a two point calibration, more elementary methods are available to account for differences in habitual levels of fatigue indicators. Example are *z*-scores based on the individual mean and SD or on the individual mean and the standard error of measurement (Kellmann et al., 2018). However, due to the reliance of standard error on the number of data points, these methods require a high number of individual measurements (a long learning phase). The downside is avoided by the gradual individualization of group-based reference ranges (Hecksteden et al., 2017).

From a physiological perspective, elucidating the reasons as well as potential practical implications of the different responses in fatigue indicators to similar training within a single training group (Figure 6) is beyond the scope of this study. However, it may be speculated that subject inherent (e.g., biological and training age, muscle fiber distribution, endurance capacity) as well as environmental factors (e.g., nutrition) play a role. The potential efficacy of changing environmental factors in minimizing training induced changes in physiological fatigue indicators (e.g., increasing carbohydrate availability during and after training to minimize protein breakdown and the subsequent increase in serum urea concentration) and ultimately in performance decrements merits further investigation. In any case, the Bayesian rationale implemented in the present method will ensure that the individualized reference range follows changes in the habitual levels of the respective fatigue marker, state, and person over time.

Importantly, transferring the diagnostic potential of advanced analytical approaches (such as individualized reference ranges) to sports practice requires adequate communication of results to athletes and coaches (Meeusen et al., 2013; Bourdon et al., 2017). The visualization of the individual “corridors” (cp. Figure 5) seems to be helpful for communicating the interpretation of measured values during the individual monitoring phase – that is for the ultimate purpose of assessing athletes fatigue status when required by the coach. For example, CK levels about 1,000 U/l after high-intensity training are habitual for player 1 but for player 2 this value would be nearly four times higher than his usual NR levels.

Formally assessing the performance of the individualization procedure during the individual monitoring phase has not been possible in this setting. This would have required either a reference classification of time points for the calculation of error rates or hard endpoints plus a large-size comparator arm to assess the ecological validity of the approach (e.g., less health problems or better performance in the experimental group in which individual reference ranges are applied). While availability of a reference classification conflicts with the aim of assessing muscle recovery at time points with (pre-test) questionable recovery status, large-scale controlled trials are impractical in an elite sport setting especially within a particular discipline and during preparation for a major event. However, based on the reductions in error rates which have now been demonstrated in different sports, assessing the ecological validity of monitoring muscle recovery based on individualized reference ranges is warranted and should be attempted in an adequately powered application study.

Exercise induced fatigue is multidimensional (Kellmann et al., 2018). Therefore, multivariate analytical tools and interpretation may be expected to be preferable. Of note, the Bayesian approach presented may be generalized to multivariate distributions. However, while this reflects the fact that exercise induced fatigue is a complex construct, the multivariate method lacks an intuitive visualization. Moreover, in a pilot implementation only minor improvements in classificatory performance could be observed (Pitsch, 2017). We have therefore intentionally decided to transfer the univariate approach.

## Limitations

Eventual benefits of monitoring muscle recovery with this method (e.g., mitigated injuries or better performance) could not be analyzed in this study due to the low subject number and lack of control group or period. This aspect warrants to be scrutinized in future studies to verify ecological validity.

The statistical method of individualizing reference ranges may be difficult to understand for the non-statistician. We never the less provide this information to ensure transparency and reproducibility. However, application of the method does not require understanding of the method on the mathematical level. The graphical representation (Figure 5 and Supplementary Figure S1) provide an intuitive plausibility control for the fitting of individual corridors. An excel spreadsheet is provided with the original publication of Hecksteden et al. (2017) and can be used with own data.

Venous blood sampling will be difficult to implement in the context of routine monitoring in high performance sports. While for this study venous blood sampling and standard automated laboratory analyses were conducted mainly to ensure similarity to the methods in the original publication, capillary sampling and mobile devices for analysis may be used for transfer into sports practice.

## Conclusion

Individual reference ranges for CK and urea seem to offer diagnostic benefits in the monitoring of muscle recovery in elite badminton, in particular when reference ranges are calculated for R and NR states offering an individual two-point calibration of the respective parameter. Visualizing the gradual development of individual “corridors” over successive measurements assists in communicating the interpretation of measured values to coaches and athletes.

## Ethics Statement

This study was carried out in accordance with the declaration of Helsinki. The protocol was approved by the local Human Research Ethics Committee (Ärztekammer des Saarlandes, approval no. 228/13 and amendments). Due to the small number of world class athletes effective anonymization of data is not possible. Therefore, data can not be published along with the manuscript.

## Author Contributions

VB conducted the blood sampling and drafted the manuscript. AH provided the notable intellectual input throughout drafting. AH and TM contributed equally as senior authors. HK was the national coach of the German Badminton Association and enabled the collaboration with the badminton players of the German national squad. AF, MK, and MP contributed to the design and funding of the study and to the interpretation of results. All co-authors drafted the manuscript and approved its final version.

## Conflict of Interest Statement

The authors declare that the research was conducted in the absence of any commercial or financial relationships that could be construed as a potential conflict of interest.

## Supplementary Material

FIGURE S1 CK reference ranges are scaled from 0 to 1250 U/l, in case of spike values the scale was adapted from 0 to 2250 U/l. Urea reference ranges are scaled from 0 to 70 mg/dl. The learning phase of each player consists of 5 R and NR values and durated approximately 6 weeks. During the monitoring phase, data were collected individually on demand of the coaches and thus players have different numbers of values. Player 10 missed measurements during the general learning phase due to illness so that monitoring phase could not be conducted before world championships. For player 14, the learning phase started 4 weeks later than for the other players and durated until shortly before the world championships.
