# Supplementary figures and images for: Individualized Monitoring of Muscle Recovery in Elite Badminton
Source: Front Physiol. 2019 Jun 26;10:778. doi: 10.3389/fphys.2019.00778 (PMC6607398; doi:10.3389/fphys.2019.00778)

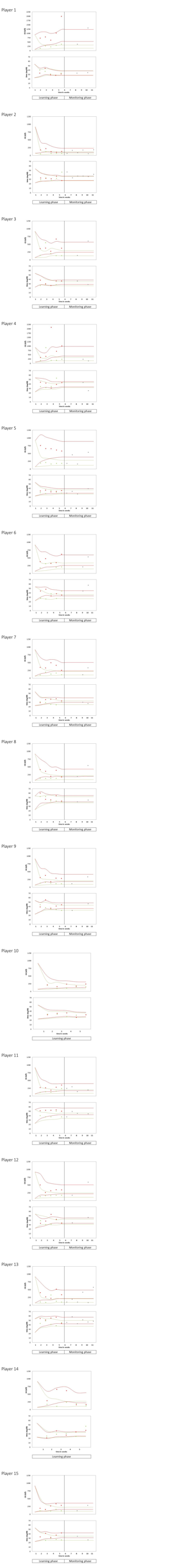

Supplement: FIGURE S1 — CK reference ranges are scaled from 0 to 1250 U/l, in case of spike values the scale was adapted from 0 to 2250 U/l. Urea reference ranges are scaled from 0 to 70 mg/dl. The learning phase of each player consists of 5 R and NR values and durated approximately 6 weeks. During the monitoring phase, data were collected individually on demand of the coaches and thus players have different numbers of values. Player 10 missed measurements during the general learning phase due to illness so that monitoring phase could not be conducted before world championships. For player 14, the learning phase started 4 weeks later than for the other players and durated until shortly before the world championships. [file Image_1.JPEG]
